# Complete mitochondrial genome and phylogenetic analysis of *Sinogastromyzon szechuanensis* (Teleostei, Cypriniformes, Homalopteridae)

**DOI:** 10.1080/23802359.2018.1443033

**Published:** 2018-02-24

**Authors:** Yuanchao Zou, Yu Yan, Mei Chen, Tian Wu, Zhengyong Wen

**Affiliations:** a College of Life Sciences, Neijiang Normal University, Conservation and Utilization of Fishes resources in the Upper Reaches of the Yangtze River Key Laboratory of Sichuan Province, Neijiang, Sichuan, China;; b School of Life Sciences, Southwest University, Chongqing, China

**Keywords:** *Sinogastromyzon szechuanensis*, mitochondrial genome, phylogenetic analysis

## Abstract

*Sinogastromyzon szechuanensis* is endemic to the Upper Yangtze River in China. In this study, we first determined the complete mitochondrial genome of *S. szechuanensis*. The circular mitogenome was 16,565 bp in length, including 13 protein-coding genes (PCGs), 2 rRNA genes, 22 tRNA genes, and 2 main noncoding regions. Most mitochondrial genes were encoded on the H-strand, except for *ND6* and eight *tRNA* genes. The overall nucleotides composition was 30.38% A, 25.26% T, 27.74% C, and 16.62% G, with AT bias of 55.64%, respectively. Phylogenetic analysis using concatenated nucleotide sequences of the 13 PCGs with two different methods (ML and NJ) both highly supported that *S. szechuanensis* showed a close relationship with *S. puliensis* and *S. sichangensis*. These data would be useful for further studies on genetic diversity and molecular phylogenetic relationship of the family Homalopteridae.

*Sinogastromyzon szechuanensis* is an endemic species to the upper reaches of Yangtze River in China (Ding 1994), belonging to the genus *Sinogastromyzon* within the subfamily Homalopterinae of the family Homalopteridae. Recent research has indicated that the natural population number of this fish strongly decreased because of overfishing and water pollution (Wu et al. 2011).

In this study, we first determined the complete mitochondrial genome of *S. szechuanensis.* The specimens were obtained from Neijiang, Sichuan province of China (30°16′17.05″N, 104°36′34.87″E) in September 2017, and were stored in Zoological Specimen Museum of Neijiang Normal University (accession number: 20170920BB03). A 30–40 mg fin clip was collected and preserved in 95% ethanol at 4 °C. Total genomic DNA was extracted with a Tissue DNA Kit (OMEGA E.Z.N.A., Norcross, GA, USA) following the manufacturer’s protocol. Subsequently, the genomic DNA was sequenced using the next-generation sequencing, and then the mitogenome was assembled using *S. sichangensis* as reference.

The complete mitochondrial genome of *S. szechuanensis* was 16,565 bp in length (GenBank Accession number MG 554420), including 13 protein-coding genes, 2 ribosomal RNA genes, 22 transfer RNA genes, an origin of light-strand replication (OL), and one displacement loop locus (D-loop). The overall nucleotides composition was 30.38% A, 25.26% T, 27.74% C, and 16.62% G, with a slight AT bias of 55.64%, respectively, which demonstrated a typical vertebrate mitochondrial genome feature (Li et al. 2012; Wang et al. 2016). Most mitochondrial genes were encoded on the H-strand except for *ND6* and eight *tRNA* genes (*tRNA^Gln^*, *tRNA^Ala^*, *tRNA^Asn^*, *tRNA^Cys^*, *tRNA^Tyr^*, *tRNA^Ser^*, *tRNA^Glu^*, and *tRNA^Pro^*). All PCGs started with ATG codon except for *COI* with GTG as start codon. Moreover, most PCGs terminated with TAA codon, except two genes (*COII*, *Cyt b*) ended with incomplete codon (T––) and one gene (*ND3*) used TAG as stop codon. Within the mitogenome of *S. szechuanensis,* there were four reading frame overlaps (ATP8-ATP6, ATP6-COIII, ND4L-ND4, ND5-ND6), ranging from 1 to 10 bp in length. The 12S rRNA and 16S rRNA were 953 and 1680 bp in length, respectively, which were located between *tRNA^Phe^* and *tRNA^Leu^* and separated by *tRNA^Val^* gene. Twenty-two tRNA genes were interspersed among the rRNAs and PCGs, ranged in size from 66 bp (*tRNA^Cys^*) to 76 bp (*tRNA^Lys^*). The D-loop was 900 bp long, and located between *tRNA^Pro^* and *tRNA^Phe^*. The origin of light-strand replication (31 bp) was located between *tRNA^Asn^* and *tRNA^Cys^*.

In order to verify the evolutionary relationship, we constructed a phylogenetic tree with the complete mitochondrial genome sequences of *S. szechuanensis* together with other 10 Homalopteridae fishes (downloaded from GenBank). Phylogenetic analysis were performed on the concatenated dataset of 13 PCGs at nucleotide level with maximum likelihood (ML) and neighbour-joining (NJ) methods (Wu et al. 2014; Wen et al. 2017; Zou et al. 2017). In addition, *Channa asiatica* and *C. argus* were defined as two outgroup species. The yielded NJ tree had a same topology as that of ML tree (Figure 1). Both trees showed that Homalopteridae was divided into two clades (A,B). Species of *S. puliensis, S. sichangensis, S. szechuanensis, Lepturichthys fimbriata, Jinshaia sinensis*, and *J. abbreviata* were clustered into clade A, and the rest of species were clustered into clade B. Additionally, both phylogenetic analyses trees supported the consanguineous relation between *S. szechuanensis, S.puliensis*, and *S.sichangensis,* which indicated these three species might own a same ancestor.

**Figure 1.**
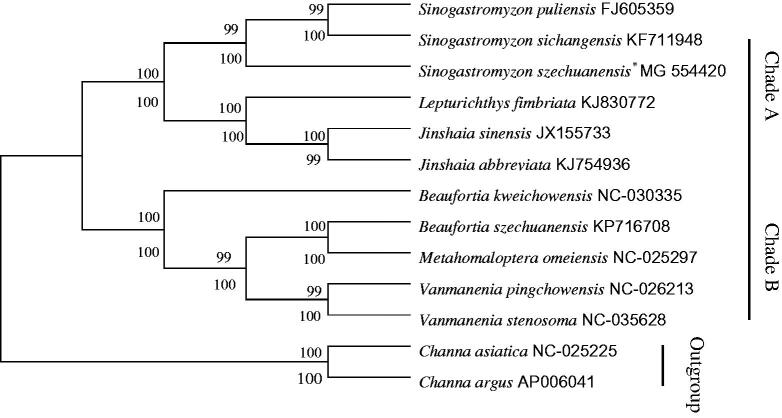
The consensus phylogenetic relationship of *S. szechuanensis* with other 10 Homalopteridae fishes. The phylogenetic analyses investigated using Neighbour-Joining (NJ) and Maximum Likelihood (ML) analysis indicated evolutionary relationships among 13 taxa based on nucleotide sequences of 13 concatenated protein-coding genes. *C. asiatica* (GenBank: NC-025225) and *C. argus* (GenBank: AP006041) were used as outgroups.
